# A phase II study evaluating the use of concurrent mitomycin C and capecitabine in patients with advanced unresectable pseudomyxoma peritonei

**DOI:** 10.1038/sj.bjc.6604522

**Published:** 2008-08-05

**Authors:** A L Farquharson, N Pranesh, G Witham, R Swindell, M B Taylor, A G Renehan, S Rout, M S Wilson, S T O'Dwyer, M P Saunders

**Affiliations:** 1 Peritoneal Tumour Service, Department of Surgery, Christie Hospital NHS Foundation Trust, Manchester, UK; 2 Department of Medical Statistics, Christie Hospital NHS Foundation Trust, Manchester, UK; 3 Department of Radiology, Christie Hospital NHS Foundation Trust, Manchester, UK; 4 School of Cancer and Imaging Sciences, University of Manchester, Manchester, UK; 5 Department of Clinical Oncology, Christie Hospital NHS Foundation Trust, Manchester, UK

**Keywords:** pseudomyxoma peritonei, chemotherapy, capecitabine, mitomycin C

## Abstract

Pseudomyxoma peritonei (PMP) is a rare neoplastic process characterised by progressive intra-abdominal dissemination of mucinous tumour, and generally considered resistant to systemic chemotherapy. A phase II study in patients with advanced unresectable PMP was undertaken to evaluate the combination of systemic concurrent mitomycin C (7 mg m^−2^ i.v. on day 1) and capecitabine (1250 mg m^−2^ b.d. on days 1–14) in a 3-weekly cycle (MCap). Response was determined by semiquantitative assessment of disease volume on serial computed tomographic (CT) scans and serum tumour marker (CEA, CA125, CA19-9) changes at 12 weeks. Between 2003 and 2006, 40 patients were recruited through a national centre for the treatment of peritoneal surface tumours. At baseline, 23 patients had progressive disease and 17 had stable disease. Of 39 assessable patients, 15 (38%, 95% confidence intervals (CIs): 25, 54%) benefited from chemotherapy in the form of either reductions in mucinous deposition or stabilisation of progressive pretreatment disease determined on CT scan. Notably, two patients, originally considered unresectable, following MCap and re-staging underwent potentially curative cytoreductive surgery. Grade 3/4 toxicity rates were low (6%, 95% CIs: 4, 9%). Twenty out of 29 assessed patients (69%, 95% CIs: 51, 83%) felt that their Global Health Status improved during chemotherapy. This is the first trial to demonstrate an apparent benefit of systemic chemotherapy in patients with advanced unresectable PMP.

Pseudomyxoma peritonei (PMP) is a rare neoplastic process (approximately 1–2 per million per year), arising in the majority of cases from the appendix, defined by disseminated peritoneal mucinous tumour deposition and progressive accumulation of mucinous ascites (Renehan *et al*, 2008). Since mid 1990s, aggressive cytoreductive surgery with removal of all visible macroscopic disease, followed by hyperthermic intraperitoneal chemotherapy (HIPEC) – often referred to as the ‘Sugarbaker’ procedure – has been promulgated as standard care (Sugarbaker, 2006). However, complete cytoreduction is possible in only one-third of cases at presentation (Rout *et al*, 2008). Disease features that prohibit complete cytoreduction include gastric encasement, diffuse small bowel mesenteric involvement and fistulation (Sugarbaker and Chang, 1999). For such advanced unresectable cases, debulking surgery may offer symptomatic relief, but is associated with considerable morbidity and mortality (Murphy *et al*, 2007; Smeenk *et al*, 2007).

Conventionally, PMP is considered resistant to systemic chemotherapy, and, to date, there are no published chemotherapy trials in the setting of advanced unresectable disease. However, a recent report described a patient with PMP responding to capecitabine – an oral fluoropyrimidine (Levitz *et al*, 2004). Given this, and the following rationales: (i) mitomycin C (MMC) and 5-fluorouracil (5-FU) are administered as HIPEC in the management of PMP (Witkamp *et al*, 2001; Sugarbaker, 2006); (ii) MMC is an established antitumour agent against gastrointestinal cancers (Crooke and Bradner, 1976; Ozawa *et al*, 1988); and (iii) oral capecitabine has equivalence effectiveness to bolus intravenous 5-FU/folinic acid in the treatment of metastatic colorectal cancer (Hoff *et al*, 2001; Van Cutsem *et al*, 2001), but with better patient acceptance (Borner *et al*, 2002), we hypothesized that a combination of concurrent MMC and capecitabine (MCap) would benefit patients with advanced PMP. Furthermore, this regimen is effective in advanced colorectal cancer (Rao *et al*, 2004).

The aim of this study was to undertake a phase II study to evaluate tumour response, survival, toxicity and quality of life in patients undergoing MCap chemotherapy for advanced unresectable PMP.

## Materials and methods

### Patients

Between April 2003 and December 2006, patients referred with histologically confirmed PMP to the Peritoneal Tumour Service of Christie Hospital NHS Foundation Trust (Manchester, UK), one of the two national centralised centres in the UK, and considered unresectable by a dedicated PMP multidisciplinary team meeting, were considered for this trial. Unresectability was determined by predefined criteria on oral contrast computed tomography (CT) scan, namely, gastric encasement, extensive small bowel mesenteric involvement and/or fistulating disease, supplemented wherever possible by pre-referral operative records and/or intraoperative photographs. Histological classification was described by Ronnett *et al* (1997),–namely, disseminated peritoneal adenomucinosis (DPAM), peritoneal mucinous carcinomatosis (PMCA) and PMCA with intermediate or discordant features (PMCA-I/D). The trial was conducted with local ethical approval, in accordance with accepted standards of good clinical practice, and in agreement with the Declaration of Helsinki.

### Inclusion and exclusion criteria

The inclusion criteria were as follows: age ⩾18 years, World Health Organisation performance status 0–2 and life expectancy >3 months. All patients were required to have adequate haematological function (neutrophil count ⩾1.5 × 10^9^ per l and platelet count ⩾150 × 10^9^ per l), hepatobiliary function (serum bilirubin ⩽1.5 × upper limit of normal (ULN); ALP ⩽5 × ULN; transaminase (AST or ALT) ⩽3 × ULN) and renal function (estimated Cockcroft clearance ⩾50 ml min^–1^). Advice about contraceptive precautions was given, and for women of childbearing potential, a negative pregnancy test was required.

The exclusion criteria were as follows: previous MMC or 5-FU-based systemic chemotherapy; concurrent uncontrolled medical illness, including cardiac disease, and previous or concurrent malignant disease; evidence of bowel obstruction; chronic diarrhoea; or if women were pregnant or lactating. Patients who had experienced life-threatening toxicities with fluoropyrimidine treatment or had any condition that might affect the absorption of capecitabine were also excluded. Patients on warfarin anticoagulation were changed to low-molecular-weight heparin (enoxaparin sodium) at the appropriate therapeutic dose.

### Treatment

**Figure 1:**
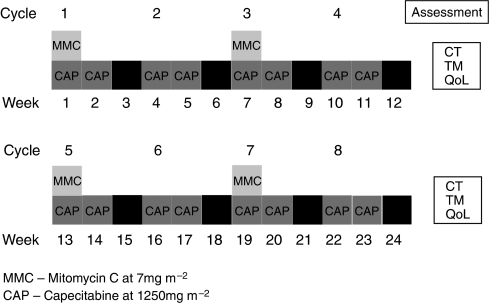
The MCap chemotherapy regimen. CT, CT scan at baseline and then 3-monthly; TM, tumour markers: CA125, CA19.9, CEA; QoL: quality of life data collected at each cycle.

The MCap regimen consisted of MMC 7 mg m^−2^ intravenous injection on day 1 and capecitabine 1250 mg m^−2^ twice daily on days 1–14, with a break in treatment on days 15–21. The next cycle consisted solely of capecitabine 1250 mg m^−2^ twice daily on days 1–14, with a break in treatment on days 15–21. These 3-week cycles were alternated so that eight cycles were given in total (four of each) (Figure 1). Prophylactic anti-emetics (ondansetron 8 mg) were administered intravenously along with MMC. Patients were treated for at least 12 weeks (4 × 3-weekly cycles) prior to response evaluation.

### Assessment of tumour response and toxicity

Patients were assessed at baseline and every 3 weeks and the following parameters recorded: performance status, weight, abdominal girth and toxicity scored using the National Cancer Institute's Common Terminology Criteria for Adverse Events version 3.0 (NCI, 2004). The following serum tumour markers were determined: carcinoembryonic antigen (CEA), cancer antigen 125 (CA125) and cancer antigen 19-9 (CA19-9).

A CT (axial computed tomography) scan was performed at baseline, and at the end of cycles 4 (12 weeks) and 8 (24 weeks). Standard CT scan RECIST criteria for the assessment of disease response were not applicable, as disease characteristics, such as cystic areas and ascites, are not included in these criteria (Padhani and Ollivier, 2001). Instead, using parallel matched computer monitors of serial CT scans, tumour response was determined semiquantitatively as either stable, reduced or progressed.

Chemotherapy was administered unless a criterion for study discontinuation was met. Treatment was stopped at the request of the patient for any reason, or if, in the opinion of the investigator, it was in the patient's best interest to do so. At each review, a score of toxicity criteria was made according to NCI-CTCAE documents. Patients were treated only if all of these toxicity criteria were grade 1 or less. Patients with persistent grade 2 symptoms were deferred until their symptoms had improved to at least grade 1. If patients experienced grade 3 or 4 toxicity, then chemotherapy was delayed until their symptoms had resolved to grade 1 or better. Further doses of capecitabine and MMC were then reduced by 20% and this was continued for the rest of the course.

### Assessment of quality of life

Quality of life was assessed using the European Organization for Research and Treatment of Cancer (EORTC) questionnaires QLQ-C30 (version 3) (Aaronson *et al*, 1993) and QLQ-CR38 (version 3) (Sprangers *et al*, 1999) at baseline, at 6 weeks and then every 12 weeks during the chemotherapy course.

### Statistical analysis

Data were collected prospectively and analysed using SPSS version 14.0® (Superior Performing Software Systems, Chicago, IL, USA). 95% confidence intervals (CIs) for single proportions were estimated using the Wilson score method without continuity correction (Newcombe, 1998). The Wilcoxon matched-pairs signed-rank test was used to compare pre- and post-treatment changes in tumour markers and quality of life data from the EORTC questionnaires QLQ C30 and CR38. Survival estimates were calculated by using the Kaplan–Meier method. Statistical significance was taken as *P*⩽0.05.

As there was no precedent, a response rate of at least 20% was considered to be acceptable in this study. Therefore, 14 patients were treated in the first instance and the study was extended on the response measured in that group. Only patients receiving the first 12 weeks of treatment were included in the assessment of the objective response rate. As more than 1 out of the first 14 patients responded, the minimum number of patients required to obtain a measurable response was 27 and the maximum 40 in order to give an estimate of the true response rate to a s.d. of 7.5%.

## Results

Forty patients were recruited to the trial. Baseline characteristics are shown in Table 1. Over one-half of the participants were female; all but one patient had undergone laparotomy elsewhere prior to referral; and two-thirds were histologically classified as DPAM. Two patients underwent laparotomy at our centre with a view to complete cytoreduction but were considered unresectable at operation and referred to the trial.

### Tumour response and survival

**Figure 2:**
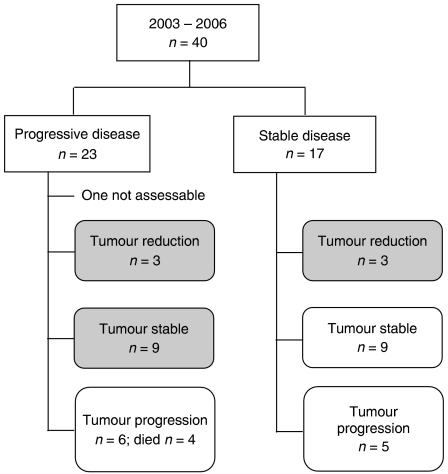
Flow diagram of treatment response. Response determined by CT scan during chemotherapy at 12 and 24 weeks and then confirmed at 6 months.

The flow diagram of disease status at baseline and subsequent responses to treatment are shown in Figure 2. Out of the 40 patients who started treatment with MCap chemotherapy, 23 (58%) had progressive disease and 17 (42%) had stable disease at baseline measurements (Figure 2).

Out of the 23 patients with progressive disease prior to trial entry, 19 patients received 3 months of treatment and 15 completed a full course of chemotherapy over 6 months. One patient had a fit during the first cycle and had no further treatment and was therefore not assessable for response. Three patients (14%) responded to the treatment with a reduction in the volume of mucus, and one of these patients also had a reduction in the volume of the solid component of their disease. After 6 months of chemotherapy, these patients had a sustained reduction in disease and nine patients (41%) were found to have stable disease. These patients had progressive disease prior to chemotherapy (Figure 2).

**Figure 3:**
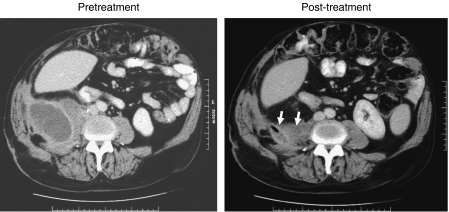
Computed tomographic scan of the abdomen at baseline and after the full treatment in a patient with initially progressive disease that shows a reduction in size of the retroperitoneal collection (arrows).

Prior to treatment, two patients had mucous discharge of approximately 50 ml day^−1^ from cutaneous fistula/sinuses. In one patient, the discharge settled completely with the cutaneous opening healing after 3 months of treatment. Another patient had two sinuses at the start of the treatment. After chemotherapy, one of the sinuses was dry and discharge from the other reduced from 50 ml day^−1^ to approximately 20 ml day^−1^. There was also a change in the consistency of the discharge from mucoid to serous (Figure 3).

Out of the 17 patients with stable disease prior to trial entry, 16 patients completed a full course of treatment. At 3 months, all 17 patients were receiving chemotherapy and were therefore assessable. Three patients (18%) responded to treatment with a reduction in the volume of mucus, which was maintained at 6 months. At this time point, nine patients (53%) were found to have stable disease and five (29%) were found to have progressive disease (Figure 2).

Overall, out of the 39 assessable patients, 15 (38%) benefited from the chemotherapy regimen in terms of a reduction in mucus (with or without a solid component) or development of stability when known to be progressing prior to treatment (Figure 2). Patients who had stable disease prior to starting treatment and had maintained stable disease during chemotherapy were considered to have received no added benefit from this therapy.

**Figure 4:**
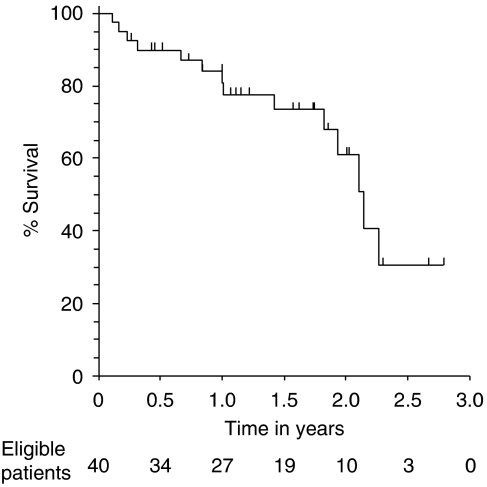
Survival curve for patients receiving MCap chemotherapy using Kaplan–Meier plot.

With a median follow up of 17 months (range 3.3–34.0), the 1-year and 2-year tumour-related survival rates for the 40 patients were 84% and 61%, respectively (Figure 4).

### Tumour marker response

**Figure 5:**
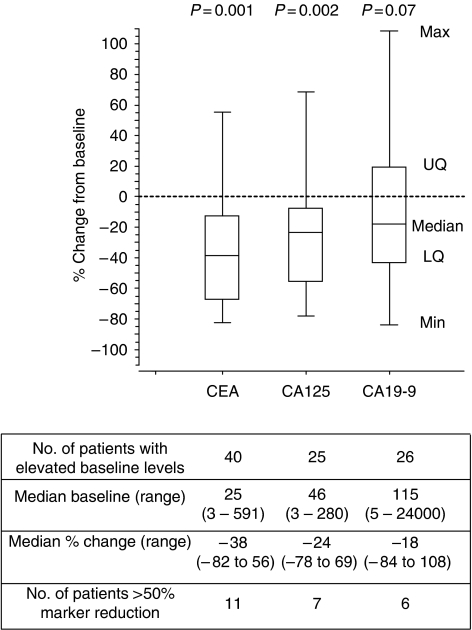
Percentage change in tumour marker level from baseline to post-treatment. Analysis limited to patients who completed a full course. CEA, carcinoembryonic antigen (normal range <3 *μ*g l^−1^); CA125, cancer antigen 125 (normal range <30 U ml^−1^); CA19-9, cancer antigen (normal range <31 U ml^−1^).

At baseline measurement, at least one serum tumour marker was raised in all patients and all three tumour markers were raised in 13 patients (33%, 95% CIs: 20, 48%). For those patients who completed a full chemotherapy course, there were reductions in the tumour marker levels by more than 50% for CEA in 11 patients; CA125 in 7 patients and CA19-9 in 6 patients. Twenty patients (51%, 95% CIs: 36, 66%) had a reduction in one or more tumour markers by more than 50% with chemotherapy. Overall, there were statistically significant reductions in the concentrations of CEA (*P*=0.001) and CA125 (*P*=0.002), but not CA19-9, between pre- and post-trial values (Figure 5). However, there were no differences in pre- and post-chemotherapy tumour marker changes when stratified by tumour response, histological type and whether progressive or stable disease at baseline. Weight, abdominal girth and performance status, which were measured at each cycle, did not alter significantly, and were not related with patient's response to treatment.

### Toxicity

There were no treatment-related mortalities; four patients (all in the progressive disease group at baseline) died early during their treatment due to disease progression; three of them had either PMCA-I/D or PMCA histological subtypes. Thirty-one patients (78%, 95% CIs: 63, 88%) completed 24 weeks of MCap chemotherapy. One patient suffered an epileptic fit during the first cycle and had no further treatment. Three patients stopped chemotherapy after the interim scans showed progressive disease; one patient stopped before the final cycle due to grade 3 toxicity. Grade 3 toxicities occurred in 12 out of 277 cycles; grade 4 toxicities in 4 out of 277 cycles – an overall rate of 6% (95% CIs: 4, 9%). All these toxicities were the hand and foot syndrome (Table 2).

### Repeat MCap chemotherapy

Despite disease progression, 17 patients had a repeat MCap chemotherapy. Of these, 7% (41, 95% CIs: 22, 64%) achieved disease stabilisation. The maximum amount of MMC given was 6 doses of 7 mg m^−2^ (42 mg m^−2^ total) – no cumulative MMC toxicity was evident. After this, patients were only treated with capecitabine at the same dose. Notably, two patients, originally considered unresectable, following MCap and re-staging underwent potentially curative cytoreductive surgery.

### Quality of life

Overall, quality-of-life parameters remained stable, with only the Global Health Status and chemotherapy-related side effects showing some alteration with treatment. The Global Health Status (QL2) provides an insight into a patients overall feeling about their well being. In this regard, out of the 15 patients with initially progressive disease prior to chemotherapy who completed a full course, 10 (67%) felt that their Global Health Status was the same or better at the end of the course, but 4 felt that it was worse (1 patient did not complete the form). Out of the 16 patients with initially stable disease prior to chemotherapy who completed a full course, 10 (63%) felt that their Global Health Status was the same or better at the end of the course, but 5 felt that it was worse (1 patient did not complete the form). Analysis of data from each section of the questionnaire showed that the only statistically significant reduction in a specific area of quality of life was chemotherapy-related side effects (*P*=0.005). There was no statistically significant difference in change in quality of life during the chemotherapy course stratified by initial disease status (stable *vs* progressive).

## Discussion

This is the first evaluation of systemic chemotherapy in patients with advanced unresectable PMP. Within the limitation of a non-randomised design, the data show that over one-third of patients appear to benefit from treatment with MCap chemotherapy without high rates of severe toxicity or reduction in quality of life.

A potential limitation of the study was the failure to quantify disease response using volume-based criteria. The role of CT scanning is well outlined for initial diagnosis. Early disease is characterised by an appendiceal tumour, and deposits predominantly on the diaphragmatic surface, around the liver and spleen and within the pouch of Douglas; as the disease progresses, deposits around the liver and spleen show scalloping and compress the adjacent organs, particularly the stomach (Sulkin *et al*, 2002). On contrast enhanced CT, PMP deposits are predominantly of low density, but may show septa and solid elements, likely to represent mucinous tumour deposits, encysted collections of mucin and fibrosis. Because of the diffuse nature of the disease, measurement of PMP disease volume is challenging. The RECIST criteria are not applicable, as these specifically exclude ascites and cystic areas as measurable lesions (Padhani and Ollivier, 2001). Changes in volume of disease within the peritoneal cavity may be assessed in some cases by measurement of relatively well-defined deposits (encysted mucin) or by measurement of the radial diameter of deposits over the surface of the liver or spleen. However, where there are diffuse deposits, volume of disease cannot be meaningfully assessed using unidimensional or bidimensional measurements. Moreover, apparent changes in the burden of intraperitoneal disease may reflect changes in the volume of mucinous ascites and not of solid tumour. The baseline scan and all subsequent scans were carried out at Christie Hospital using the same protocol. All scans were reported by radiologists who are experienced in the radiological evaluation of PMP.

In an effort to overcome these potential limitations, CT assessment of treatment was approached systematically using three criteria: (i) overall assessment of the disease volume including mucin (supported by measurement of discrete deposits where possible); (ii) recording of new disease sites and (iii) the extent of compressive effects of disease on intraperitoneal organs, for example, degree of scalloping of the liver and spleen, compression of the stomach and strictures of the small or large bowel. Decreases in volumes of mucin alone were not recorded as responses.

The present study has a number of advantages. First, data on treatment responses, survival, toxicity and quality of life were collected prospectively. Second, uniform criteria were used to report histological classifications and radiological responses. Third, the study was set within the framework of a dedicated Peritoneal Tumour Multidisciplinary meeting (Rout *et al*, 2008), which assesses resectability and directs patients along appropriate clinical pathways. This achievement was brought about through the establishment of two centralised national treatment services – Christie Hospital NHS Foundation Trust (Manchester, UK) and North Hampshire Hospitals NHS Foundation Trust (Basingstoke, UK) (Moran, 2006)–commissioned by the UK National Health Service (NHS) National Commissioning Group for Highly Specialised Services. This funding system circumnavigates many logistic problems of undertaking a trial in a rare tumour.

We explored whether serum tumour markers, namely CEA, CA125 and CA19-9, may be helpful in the prediction of response to systemic chemotherapy. We rationalised the following: (i) serum CEA and CA19-9 levels are elevated in over 50% PMP patients at presentation and drop markedly following cytoreductive surgery and HIPEC (van Ruth *et al*, 2002); (ii) elevated CA 19-9 levels prior to cytoreductive surgery may be an independent predictor of worse progression-free survival (Baratti *et al*, 2007); and (iii) in a case study of a patient with DPAM treated with capecitabine, there was a decrease in CEA and CA19-9 levels (Levitz *et al*, 2004). Serum CA125 concentrations are commonly raised in patients with PMP, and at initial presentation, may mimic gynaecological malignancies (Pranesh *et al*, 2005). However, it is noteworthy that serum CA125 levels may also be raised in conditions including peritoneal irritation (Sjovall *et al*, 2002), pelvic inflammatory disease (Halila *et al*, 1986) and benign hepatic disease (Topalak *et al*, 2002). Although our study demonstrated elevated levels of all three markers in one-third of the cases at baseline, and reductions of 50% or more in half of these, there was no relationship between treatment response and decrease in marker level. Although the present study is relatively small, tumour marker response does not appear helpful in determining chemotherapy response.

For an unselected population of patients with PMP, over half of the cases at initial presentation will be advanced and unresectable. For these, we recommend 3-monthly CT scan. Where disease is stable, a ‘watch and wait’ policy with repeat imaging every 3–6 months may be prescribed. These patients should be considered for MCap chemotherapy if they become symptomatic or their disease is radiologically progressing. We recommend up to a 6-month course of treatment followed by a rest period with CT surveillance; repeat MCap courses may be prescribed if disease progresses (the maximum total dose of MMC used in this study was 42 mg m^−2^).

One unresolved questions from this study is that of optimising patient selection criteria. We noted that three out of the four patients that died early had aggressive histological types. Based on its effectiveness in the management of advanced colorectal cancer (Saunders and Iveson, 2006), we hypothesize that the use of oxaliplatin and 5-FU combination may be beneficial in such cases. This regimen has yet to be systematically tested and will have to address additional practical issues, such as the requirement for central venous access and cumulative toxicity such as parasthesia. Although the understanding of the basic biology of PMP is still in its infancy (Bibi *et al*, 2006), for the immediate future, systemic antitumour approaches in the treatment of advanced unresectable disease will probably emerge from existing strategies for related gastrointestinal malignancies.

## Figures and Tables

**Table 1:** Baseline patient characteristics

	***n*^a^ (%)**
Patients	40
Median age (range), years	59 (32–77)
Males/females	12 : 28
	
*Histological group*	
DPAM	27 (68)
PMCA-I/D	10 (25)
PMCA	3
	
*Previous laparotomies*	
No previous laparotomies	1
1	22 (55)
2	12 (30)
⩾3	5
	
*WHO performance status*	
0	36 (90)
1	2
2	2

DPAM=disseminated peritoneal adenomucinosis; PMCA=peritoneal mucinous carcinomatosis; PMCA-I/D=PMCA with intermediate or discordant features.

a Number of patients unless otherwise stated.

**Table 2:** Number and grade of toxicity events at each cycle during course

**Cycle**	**1**			**2**			**3**			**4**			**5**			**6**			**7**			**8**		
**Evaluable patients**	**40**			**38**			**36**			**35**			**34**			**32**			**32**			**30**		
NCI-CTCAE grade	2	3	4	2	3	4	2	3	4	2	3	4	2	3	4	2	3	4	2	3	4	2	3	4
Alopoecia																								
Anorexia	1																							
Constipation																						1		
Diarrhoea				3			1				2		1									1		
HFS							3	2		4	4		3				1	4	1			7		
Lethargy	5	1		7			9			8			6			6			7					
Nausea	1	1		2			4			1			1						1					
Neuropathy																								
Stomatitis							2			1			1											
Vomiting		1		1						2									1			7		

HFS=hand and foot syndrome; NCI-CTCAE v3.0=National Cancer Institute Common Toxicity Criteria for adverse events.
